# The dirigent multigene family in *Isatis indigotica*: gene discovery and differential transcript abundance

**DOI:** 10.1186/1471-2164-15-388

**Published:** 2014-05-20

**Authors:** Qing Li, Junfeng Chen, Ying Xiao, Peng Di, Lei Zhang, Wansheng Chen

**Affiliations:** Department of Pharmacy, Shanghai Changzheng Hospital, Second Military Medical University, No. 415 Fengyang Road, Huangpu District, Shanghai, 200003 China; Department of Pharmaceutical Botany, School of Pharmacy, Second Military Medical University, No. 325 Guohe Road, Yangpu District, Shanghai, 200433 China; The MOE Key Laboratory of Standardization of Chinese Medicines, Institute of Chinese Materia Medica, Shanghai University of Traditional Chinese Medicine, Shanghai, 201210 China

**Keywords:** Dirigent and dirigent-like proteins, *Isatis indigotica*, Bioinformatics, Secondary structures, Tertiary structures, Phylogenetic analysis, Transcript abundance

## Abstract

**Background:**

*Isatis indigotica* Fort. is one of the most commonly used traditional Chinese medicines. Its antiviral compound is a kind of lignan, which is formed with the action of dirigent proteins (DIR). DIR proteins are members of a large family of proteins which impart stereoselectivity on the phenoxy radical-coupling reaction, yielding optically active lignans from two molecules of *E*-coniferyl alcohol. They exist in almost every vascular plant. However, the DIR and DIR-like protein gene family in *I. indigotica* has not been analyzed in detail yet. This study focuses on discovery and analysis of this protein gene family in *I. indigotica* for the first time*.*

**Results:**

Analysis of transcription profiling database from *I. indigotica* revealed a family of 19 full-length unique DIR and DIR-like proteins. Sequence analysis found that *I. indigotica* DIR and DIR-like proteins (*Ii*DIR) were all-beta strand proteins, with a signal peptide at the *N*-terminus. Phylogenetic analysis of the 19 proteins indicated that the *Ii*DIR genes cluster into three distinct subfamilies, DIR-a, DIR-b/d, and DIR-e, of a larger plant DIR and DIR-like gene family. Gene-specific primers were designed for 19 unique *Ii*DIRs and were used to evaluate patterns of constitutive expression in different organs. It showed that most *Ii*DIR genes were expressed comparatively higher in roots and flowers than stems and leaves.

**Conclusions:**

New DIR and DIR-like proteins were discovered from the transcription profiling database of *I. indigotica* through bioinformatics methods for the first time. Sequence characteristics and transcript abundance of these new genes were analyzed. This study will provide basic data necessary for further studies.

**Electronic supplementary material:**

The online version of this article (doi:10.1186/1471-2164-15-388) contains supplementary material, which is available to authorized users.

## Background

*Isatis indigotica* Fort. is one of the most commonly used plants in traditional Chinese medicine for its anti-inflammatory and antiviral activities [1]. Its leaves are called “Daqingye” (*Folium Isatidis*), which can be used for the treatment of high fever, epidemic parotitis, pharyngitis and erysipelas. The root of *I. indigotica* is the well-known Chinese medicine “Banlangen” (*Radix Isatidis*), which is widely used for flu and infections of the upper respiratory tract in China. During the epidemic period of severe acute respiratory syndromes (SARS) in 2003, Banlangen demonstrated the potential prevention of SARS [2]. However, the antiviral compounds of *I. indigotica* were still unknown until Li [3] learned that lariciresinol isolated from this plant was useful for the treatment of influenza A1 virus.

Lariciresinol is a kind of lignan which has been widely studied and reported to possess a number of biological activities, including antimicrobial, antioxidant, anti-inflammatory and anti-estrogenic properties, which may reduce the risk of cardiovascular diseases, as well as certain types of cancer [4–9]. The precursor of lariciresinol is pinoresinol, which comes from *E*-coniferyl alcohol by the action of dirigent proteins (DIR) [10].

Dirigent (Latin: *dirigere*, to guide or align) proteins are members of a large family which imparts stereoselectivity on the phenoxy radical-coupling reaction. These proteins can capture *E*-coniferyl alcohol (only *E*-coniferyl alcohol, not *p*-coumaryl or sinapyl alcohols which differ only in the degree of aromatic methoxylation [10]) derived free-radical intermediates and orientate these radicals in such a way as to enable 8-8’ coupling with concomitant intramolecular cyclization to afford optically active (+)- or (−)-pinoresinol [11–14]. In the absence of DIR proteins, only non-specific radical-radical coupling occurs at the 8-8’, 8-5’, or 8-O-4’ positions with the resulting formation of racemic lignan products [12–14].

DIR proteins exist in almost every vascular plant [15]. Ralph and coworkers [16] suggest that the DIR proteins are subdivided into five groups: the DIR-a, DIR-b, DIR-c, DIR-d and DIR-e subfamilies. With the increasing numbers of DIR proteins, the DIR-b and DIR-d subfamilies are combined together with the appearance of the DIR-f and DIR-g subfamilies [17]. However, only members of DIR-a subfamily are being studied for their biochemical functions; the other proteins are referred to as DIR-like proteins. The DIR and DIR-like protein gene family in *I. indigotica* has not been analyzed in detail yet. Under the umbrella of a transcription profiling of *I. indigotica*[18], 19 full-length *IiDIRs* (the dirigent or dirigent-like protein genes of *I. indigotica*) are mined analytically through bioinformatics. Here we report an inventory and sequence analysis as well as the phylogenetic relationships of the *Ii*DIR gene family. A detailed quantitative real-time PCR expression analysis in constitutive *I. indigotica* tissues is described for 19 *Ii*DIRs. Finally, we provide a transcriptome analysis of *Ii*DIRs, which is based on data treated with MeJA at different time points.

## Results

### Discovery of *IiDIRs* from the *I. indigotica* transcription profiling database

Using TBLASTN and BLASTN (Basic Local Alignment Search Tool 2.2.26) against the *I. indigotica* transcription profiling database with released DIR and DIR-like protein sequences, we obtained 19 putative *IiDIR* sequences (Additional file 1). The best hit homology genes of these 19 sequences were summarized in Additional file 2. The number and subfamily designation of the *IiDIR* genes were based on the topology of the 19 *IiDIRs* with other 178 *DIRs* according to Ralph [17] and Arasan [19]. Typical dirigent domains were found in these 19 *IiDIR* protein sequences though simple modular architecture research tool (SMART, http://smart.embl-heidelberg.de/) [20] (Additional file 3).

### Sequences analysis

The length of the predicted open reading frames (ORFs) for the 19 cDNAs ranged from 183 aa (*Ii*DIR1) to 414 aa (*Ii*DIR19). The 19 *Ii*DIRs had predicted molecular masses range from circa 20.17 (*Ii*DIR8) to 39.94 (*Ii*DIR19) kDa and predicted *p*I values range from 4.79 (*Ii*DIR16) to 9.85 (*Ii*DIR8) (Additional file 3).

Using the TargetP 1.1 Server (http://www.cbs.dtu.dk/services/TargetP/) [21] and the WoLF PSORT (http://www.genscript.com/psort/wolf_psort.html) [22] subcellular localization software, it was predicted that most of the 19 *Ii*DIRs were targeted to the secretory pathway, either through the default pathway for extracellular release, or for possible final localization in the vacuolar, chloroplast and cytoplasmic locations. The signal peptide prediction showed that most of the *Ii*DIR*s* had a 20–30 aa length signal peptide at the *N*-terminus except *Ii*DIR13, *Ii*DIR15, and *Ii*DIR18. All *Ii*DIRs except *Ii*DIR12/13/14/15/16/17 were found to contain N-glycosylation sites (Asn) which were a feature of secreted proteins using NetNGlyc 1.0 server (http://www.cbs.dtu.dk/services/NetNGlyc/) [23] (Additional file 3). The SMART results showed that *Ii*DIR3/4/12/13/14/15/17/19 had transmembrane region. The molecular formula was calculated through ProtParam (http://web.expasy.org/protparam/) [24]. It found out that, the gene with the most sulfur elements was *IiDIR*11 (12 sulfur elements), while *IiDIR*16 and *IiDIR*17 only had three sulfur elements, respectively (Additional file 3).

Pairwise sequence similarities among predicted amino acids of the19 *Ii*DIRs ranged from a low of 14.4% identity (*Ii*DIR4 vs. *Ii*DIR13, *Ii*DIR14, *Ii*DIR15, respectively) to a high of 98.1% (*Ii*DIR14 vs. *Ii*DIR15) (Table 1). *Ii*DIR14 and *Ii*DIR15 were an example of closely related proteins sharing amino acid identity greater than 98% that may represent within-species alleles.

**Table 1:** **Sequence relatedness of** ***Ii*** **DIRs**

	a	b	c	d	e	f	g	h	i	j	k	l	m	n	o	p	q	r	s
*Ii*DIR1(a)	-																		
*Ii*DIR2(b)	76.9	-																	
*Ii*DIR3(c)	63.8	70.0	-																
*Ii*DIR4(d)	65.0	69.4	90.0	-															
*Ii*DIR5(e)	*25.0*	*24.4*	*20.6*	*19.4*	-														
*Ii*DIR6(f)	*25.6*	*24.4*	*20.6*	*19.4*	91.9	-													
*Ii*DIR7(g)	*25.6*	*26.3*	*23.8*	*22.5*	63.1	66.3	-												
*Ii*DIR8(h)	*25.0*	*25.6*	*23.1*	*21.9*	62.5	66.3	96.3	-											
*Ii*DIR9(i)	*21.3*	*20.0*	*18.8*	*17.5*	39.4	40.6	45.0	43.8	-										
*Ii*DIR10(j)	*18.1*	*19.4*	*19.4*	*18.8*	41.3	41.9	45.6	44.4	71.3	-									
*Ii*DIR11(k)	*22.5*	*26.3*	*22.5*	*20.6*	44.4	45.6	43.8	43.8	41.9	46.3	-								
*Ii*DIR12(l)	*18.1*	*18.8*	*14.4*	*15.6*	19.4	20.0	20.6	21.3	18.8	17.5	19.4	-							
*Ii*DIR13(m)	***17.5***	***20.0***	***15.6***	***14.4***	**23.1**	**23.1**	**23.1**	**23.8**	**20.6**	**21.3**	**20.6**	**16.9**	-						
*Ii*DIR14(n)	***17.5***	***19.4***	***15.6***	***14.4***	**23.8**	**23.8**	**23.8**	**24.4**	**21.9**	**22.5**	**21.3**	**17.5**	95.6	-					
*Ii*DIR15(o)	***17.5***	***19.4***	***15.6***	***14.4***	**23.8**	**23.8**	**23.8**	**24.4**	**21.3**	**21.9**	**21.3**	**17.5**	97.5	98.1	-				
*Ii*DIR16(p)	***20.0***	***20.6***	***17.5***	***16.9***	**24.4**	**24.4**	**25.6**	**24.4**	**23.8**	**23.1**	**26.9**	**20.0**	41.3	41.3	41.9	-			
*Ii*DIR17(q)	***18.1***	***18.8***	***15.6***	***15.0***	**21.3**	**23.8**	**23.8**	**22.5**	**22.5**	**21.3**	**25.6**	**18.8**	42.5	42.5	43.1	90.0	-		
*Ii*DIR18(r)	***15.6***	***18.8***	***16.9***	***16.3***	**25.0**	**23.1**	**26.9**	**25.6**	**24.4**	**25.6**	**24.4**	**16.9**	50.6	50.6	50.6	55.0	56.3	-	
*Ii*DIR19(s)	***15.6***	***18.8***	***15.6***	***15.0***	**24.4**	**22.5**	**26.3**	**25.0**	**23.1**	**23.1**	**25.6**	**16.3**	51.9	51.9	51.9	54.4	55.6	91.9	-

Results from pairwise amino acid sequence comparisons, using complete open reading frames, show as percent identity among members of the DIR-a, DIR-b/d and DIR-e subfamilies. Comparisons within the subfamily are printed as normal, whereas comparisons between proteins of the subfamilies DIR-a and DIR-b/d, DIR-a and DIR-e, and DIR-b/d and DIR-e are highlighted in italic, underline, and bold, respectively.

### Secondary structures of *Ii*DIRs

Additional file 4 presented the secondary structures polygrams of the 19 *Ii*DIRs. They were predicted by NetSurfP (http://www.cbs.dtu.dk/services/NetSurfP/) [25]. The 19 *Ii*DIR proteins could be divided into several groups according to their secondary structures. The differences among all the *Ii*DIRs were existing in the residues before the first β-strand. According to this shape, *Ii*DIR16/17/18/19 were far away from *Ii*DIR1 to *Ii*DIR15. In the first 15 *Ii*DIRs, *Ii*DIR13/14/15 were different from others at the shape of region between the first β-strand and the second β-strand. *Ii*DIR12 was different from others at the last shape of β-strand. *Ii*DIR1/2/3/4/were different from other *Ii*DIRs because they had a smooth Coil curve before the first β-strand.

To confirm the forecast accuracy of NetSurfP, predictions of the secondary structures were also carried out on PSIPRED (http://bioinf.cs.ucl.ac.uk/psipred/) and Phyre2 (http://www.sbg.bio.ic.ac.uk/phyre2/html/page.cgi?id=index) [26]. The position of the β-strands was in overall agreement with the predictions determined by NetSurfP (data not shown).

### Tertiary structures and homologs of *Ii*DIRs

**Figure 1:**
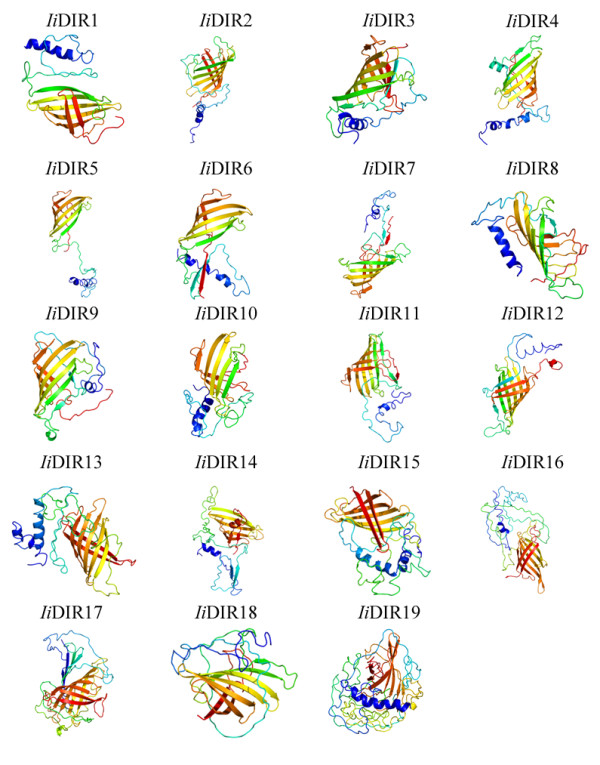
**Cartoon-style model of** ***Ii*** **DIRs derived from prediction.**

Figure 1 showed predicted three-dimensional structures of 19 *Ii*DIR proteins. Structures of the 19 proteins were modeled using the server: http://www.sbg.bio.ic.ac.uk/phyre2/html/page.cgi?id=index[26]. For all of the19 queried sequences, the same three top-scoring proteins were found, all of which belong to the allene oxide cyclase-like protein (AOC) family. AOC barrel-like protein d2brja1, which shared only 17–26% sequence identity among the *Ii*DIRs, was predicted as a DIR homolog with about 98% probability, followed by two hypothetical proteins with similar probabilities (d1zvca1 and c4h69A, Table 2). Among the highest confidence level predicted by Phyre2, *Ii*DIR14 and *Ii*DIR18 showed the highest confidence of 98.3% respectively with the template d2brja1.

**Table 2:** **The probability and identity of homologous relationship of** ***Ii*** **DIRs**

	d2brja1		c4h69A		d1zvca1	
	% Confidence	% Identity	% Confidence	% Identity	% Confidence	% Identity
*Ii*DIR1	97.8	21	97.7	21	97.7	22
*Ii*DIR2	97.9	21	97.6	22	97.6	21
*Ii*DIR3	97.9	20	97.7	18	97.8	20
*Ii*DIR4	98.0	20	97.6	19	97.6	20
*Ii*DIR5	97.8	22	97.8	21	97.7	24
*Ii*DIR6	97.8	21	97.8	20	97.7	24
*Ii*DIR7	97.8	18	97.7	18	97.7	19
*Ii*DIR8	97.8	17	97.7	19	97.7	19
*Ii*DIR9	97.8	21	97.4	18	97.5	19
*Ii*DIR10	97.8	20	97.7	19	97.7	20
*Ii*DIR11	97.8	22	97.8	23	97.8	22
*Ii*DIR12	98.1	19	97.6	20	97.7	18
*Ii*DIR13	98.0	25	97.9	22	97.9	22
*Ii*DIR14	98.3	25	98.2	21	98.2	24
*Ii*DIR15	98.0	25	97.9	22	97.9	23
*Ii*DIR16	97.9	21	97.7	21	97.7	24
*Ii*DIR17	98.2	20	98.1	21	98.2	21
*Ii*DIR18	98.3	26	98.1	25	98.3	24
*Ii*DIR19	97.9	22	97.9	25	97.9	24

### Phylogenetic analysis of the *Ii*DIRs

**Figure 2:**
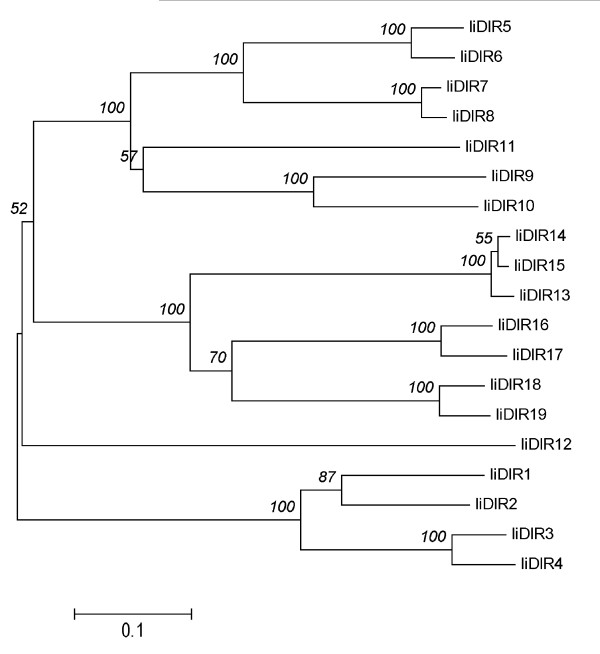
**Neighbor-Joining (NJ) phylogenetic trees of 19** ***Ii*** **DIRs.** The values on the branches are bootstrap proportions, which indicated the percentage values for obtaining this particular branching in 1000 repetitions of the analysis. The lengths of branches are proportional to evolutionary distances between species.

To obtain clues about the evolutionary relationships and the topological structures of the *Ii*DIRs, multiple sequence alignments of amino acid sequences of the 19 full-length cDNAs were used to build a Neighbor-Joining (NJ) tree with 1000 bootstrap reconstruction and completed deletion gaps/missing data treatment (Figure 2). The 19 *Ii*DIRs were clearly separated into three distinct groups based on sequence relatedness. The amino acid sequences of *Ii*DIR1/2/3/4 were clustered into Group 1, while *Ii*DIR5/6/7/8/9/10/11 were clustered into the second group. These two groups were in accordance with the secondary structures. *Ii*DIR13/14/15/16/17/18/19 were clustered into another group. *Ii*DIR 12 was left behind between Group 1 and other *Ii*DIRs.

To test the reliability of the NJ tree, a Maximum Likelihood (ML) analysis was also carried out to generate a phylogenetic tree using default parameters and 1000 bootstrap reconstruction as well (Additional file 5, −ln = 3970.33, model: WAG + F). Both of the two trees had similar topological structures with three clusters, which indicated that the two methods were in good agreement.

**Figure 3:**
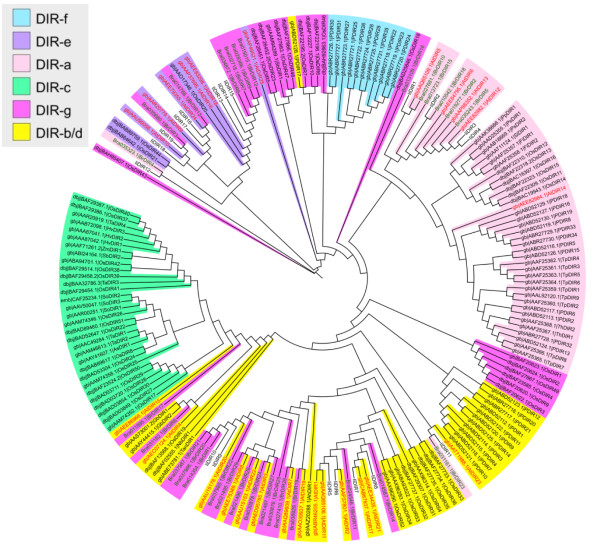
**Phylogenetic tree of plant DIR and DIR-like protein sequences.** Amino acids of 197 dirigent or dirigent-like (DIR) proteins are analyzed by Maximum Likelihood (ML) using MEGA 5.05 (−ln = 2880.02, model: WAG + F). Subfamilies DIR-a, DIR-b/d, DIR c, DIR-e, DIR-f and DIR-g are indicated by pink, yellow, green, purple, skyblue and pink-purple shading respectively. The *At*DIRs are colored in red and *Br*DIRs are colored in darkgreen. *Ii*DIRs are marked as normal. DIR nomenclature is as follows: Ah, *Arachis hypogaea*; As, *Agrostis stolonifera*; At, *Arabidopsis thaliana*; Br, *Brassica rapa*; Fi, *Forsythia intermedia*; Gb, *Gossypium barbadense*; Hv, *Hordeum vulgare*; Ii, *Isatis indigotica;* Nb, *Nicotiana benthamiana*; Os, *Oryza sativa*; P, *Picea glauca*, *Picea sitchensis* or *P. glauca x engelmannii*; Pp, *Podophyllum peltatum*; Ps, *Pisum sativum*; Sb, *Sorghum bicolor*; Si, *Sesamum indicum*; So, *Saccharum officinarum*; Ta, *Triticum aestivum*; Tan, *Tamarix androssowii*; Th, *Tsuga heterophylla*; Tp, *Thuja plicata* and Zm, *Zea mays*.

To better understand DIR and DIR-like protein sequences divergences and similarities among *I. indigotica* and other plants, a provisional molecular phylogenetic tree was constructed using multiple sequence alignment from various plant species. These gene sequences were as follows: 29 genes from *Brassica rapa*, 25 genes from *Arabidopsis thaliana*, 54 genes from *Oryza sativa*, 35 genes from spruce, 9 genes from *Thuja plicata*, and an additional 27 DIRs identified from a variety of species, including pea, cotton, corn, sesame, etc. [17, 19]. In this tree, different subfamilies according to Ralph [17] were colored in different colors. However, only DIR-a, DIR-c and DIR-f subfamilies were clustered separately. DIR-e and DIR-b/d subfamilies were mixed with genes from DIR-g subfamily. These DIR-g subfamily genes were all from *B. rapa.* The phylogenetic tree indicated that *Ii*DIRs cluster into three groups, DIR-a, DIR-b/d and DIR-e (Figure 3). *Ii*DIR1/2/3/4 grouped into subfamily DIR-a, along with 5 *A. thaliana* genes and 6 *B. rapa* genes. *Ii*DIR5/6/7/8/9/10/11 grouped into subfamily DIR-b/d, along with 14 *A. thaliana* genes and 16 *B. rapa* genes. *Ii*DIR13/14/15/16/17/18/19 grouped into subfamily DIR-e, along with 6 *A. thaliana* genes and mixed with 4 *Br*DIRs from DIR-g subfamily. *Ii*DIR 12 was outside the subfamily DIR-e. We designated it to subfamily DIR-e.

### Sequence comparison

The *Ii*DIR sequences were analyzed to address if any putative functions could be inferred. The topology analyses of the *Ii*DIRs showed that they contribute to DIR-a, DIR-b/d, and DIR-e subfamilies. Recent studies only focused on the function of the DIR-a subfamily, classifying the other DIRs from the other subfamilies to be the DIR-like proteins. According to Pickel [11, 27], *At*DIR5 and *At*DIR6 from DIR-a subfamily of *A. thaliana* were different from those DIRs found earlier, such as *Fi*DIR1 and *Tp*DIR7 [28]. The first DIR from *Forsythia suspensa* was found to guide *E*-coniferyl alcohol to form (+)-pinoresinol [12], and many other DIRs had the same function [29]. However, in the presence of *At*DIR6, the final product of *E*-coniferyl alcohol was the enantiomer (−)-pinoresinol. From the topology tree of DIRs from different species (Figure 3), *Ii*DIR2/3/4 were adjacent to *At*DIR6, and *Ii*DIR1 was next to *At*DIR5. These observations suggested that *Ii*DIR1/2/3/4 might have similar functions with *At*DIR6 and *At*DIR5.

**Figure 4:**
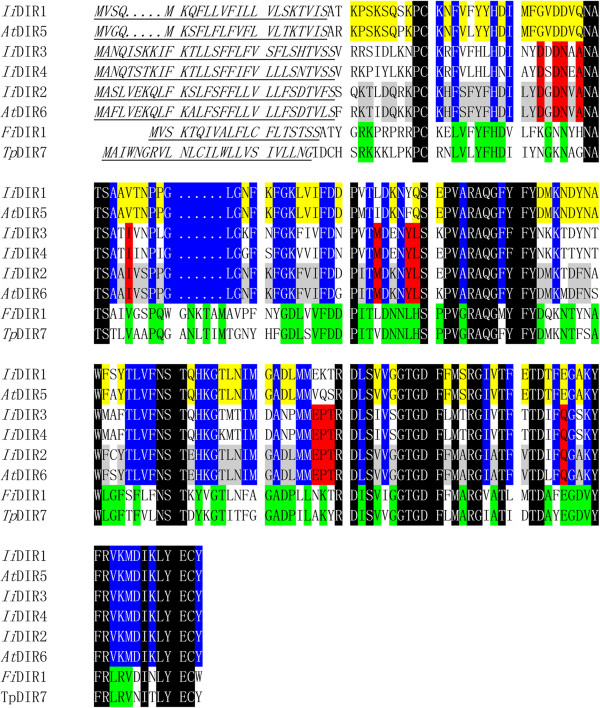
**Sequence comparison between DIRs from** ***Forsythia intermedia*** **,** ***Thuja plicata*** **,** ***Arabidopsis thaliana*** **and** ***Isatis indigotica.*** Residues conserved in all of the sequences are indicated in black. Sequence conservation between *A. thaliana* and *I. indigotica* is highlighted in blue. Conservation between *T. plicata* and *F. intermedia* is highlighted in green. Conservation between *At*DIR6, *Ii*DIR2, *Ii*DIR3 and *Ii*DIR4 is highlighted in red. Conservation between *At*DIR5 and *Ii*DIR1 is highlighted in yellow. Conservation between *At*DIR6 and *Ii*DIR2 is highlighted in gray. Predicted *N*-terminus signal peptides are shown in italics with underline.

Sequence comparisons between *At*DIR6 and *Ii*DIR2/3/4 as well as *At*DIR5, *Ii*DIR1, *Fi*DIR1 and *Tp*DIR7 were performed by clustalX 2.1 [30]. The results showed that *Ii*DIR2 had 93.05% identity with *At*DIR6, while *Ii*DIR3 and *Ii*DIR4 had only 68.45% and 66.31% identity with *At*DIR6. It suggested that the relative among *Ii*DIR3, *Ii*DIR4 and *At*DIR6 might far away from that between *Ii*DIR2 and *At*DIR6. *Ii*DIR1 had 90.66% identity with *At*DIR5. Residues conservation was shown in Figure 4.

**Figure 5:**
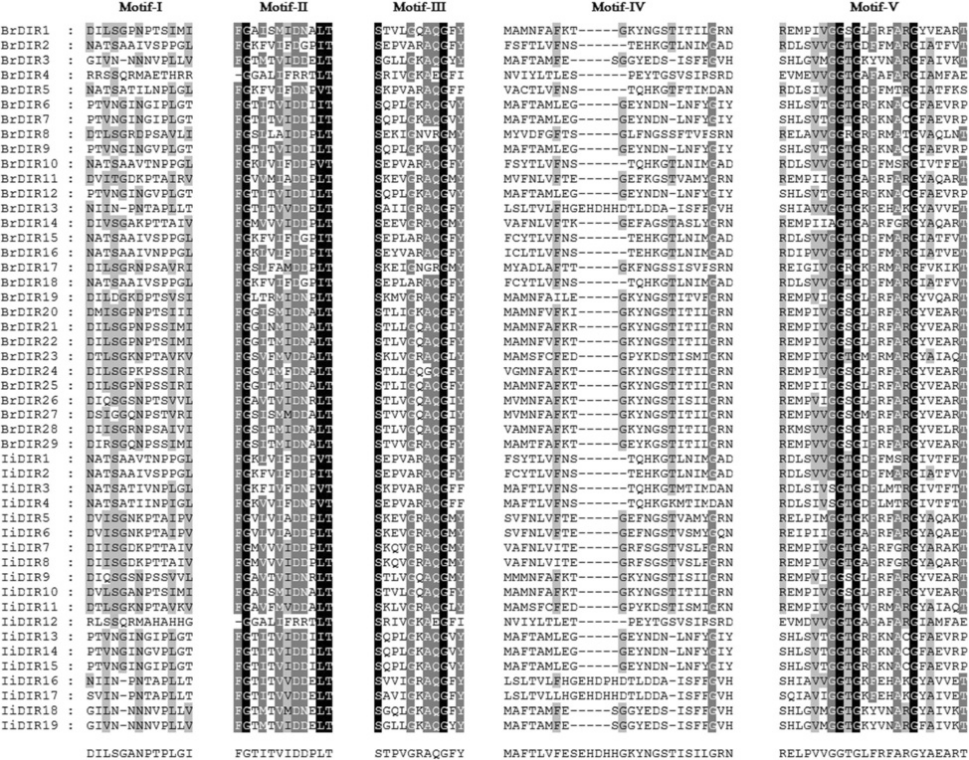
**Conserved five characteristic motifs (I-V) of dirigent proteins in** ***Ii*** **DIR and** ***Br*** **DIR protein sequences.**

To examine sequence features of these *Ii*DIR sequences, sequence comparison between 19 *Ii*DIRs and 29 *Br*DIRs were carried out as well. The 19 *Ii*DIRs showed five well conserved motifs in their amino acid sequences like 29 *Br*DIRs (Figure 5).

### Transcript abundance analysis of *Ii*DIRs in different organs

**Figure 6:**
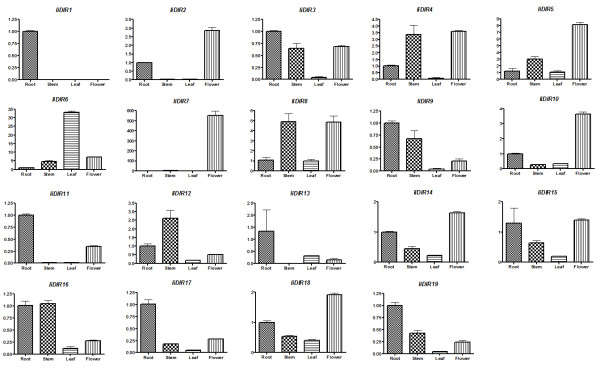
**Quantitative real-time PCR analysis of constitutive** ***IiDIR*** **s transcript abundance in different organs.** Transcript abundance of each *Ii*DIR gene is normalized to *actin* as control and compared with root as reference using 2^-△△Ct^ method. Values obtained by real-time PCR represent mean ± SEM (n = 3).

Since the transcript abundance of a gene was often correlated with its function, the relative constitutive abundance of the 19 *IiDIRs* were quantified in total RNA isolated from roots, stems, leaves and flowers through real-time PCR using gene-specific primers (Additional file 6). The organ specific expression of each *IiDIR* gene was normalized to *actin* as control and compared with root as reference using 2^-△△Ct^ method. The transcript abundance level was showed in Figure 6.

Based on RT-PCR analysis, 5 *IiDIR*s (*IiDIR*2/5/10/15/18) displayed the highest transcript abundance in all tissues. Another 5 *IiDIR*s (*IiDIR*3/4/11/13/17) showed higher transcript abundance in roots and flowers than in stems and leaves. *IiDIR*6 was higher in leaves and *IiDIR*7 was higher in flowers. *IiDIR*1/12/13/16/19 were hardly expressed in leaves. The remaining two *IiDIR*s (*IiDIR*8 and *IiDIR*9) were nearly not detected in any tissue (Additional file 7).

Compared with the gene transcript abundance in roots, *IiDIR*7 was more than 500 fold higher in flowers. All of these genes were lowly expressed in leaves than in other tissues except *IiDIR*6. Most *IiDIRs* have comparatively higher transcript abundance in roots and flowers than in stems and leaves, such as *IiDIR*2/10/11/14/15/17/18. The transcript abundance of *IiDIR*4 and *IiDIR*8 were higher in stems and flowers than in roots and leaves. *IiDIR*12 and *IiDIR*16 were expressed more in stems (Figure 6).

### Transcript abundance analysis after treatment with MeJA

**Figure 7:**
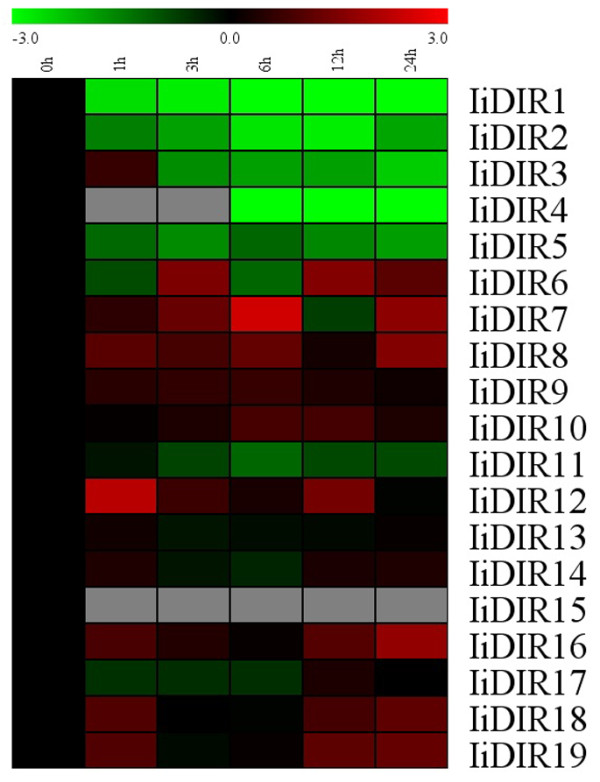
**Heat map of** ***Ii*** **DIR transcript expression obtained after treatment with MeJA at hairy roots.** A color bar indicates fold-change expression differences on a natural log scale (treatment/control). Hairy roots of *I. indigotica* are treated with MeJA for 0, 1, 3, 6, 12 and 24 h. 0 h is designed as control.

MeJA was used to induce the gene transcript abundance at hairy roots of *I indigotica* for different times. The *IiDIRs*’ transcript abundance was showed in Figure 7. The result of *IiDIR*15 was not tested during this experiment. *IiDIR*1/2/4/5/11 were down regulated at 1, 3, 6, 12 and 24 h compared with 0 h. *IiDIR*8/9/10/16 were up regulated at 1, 3, 6, 12 and 24 h compared with 0 h. The left genes were up or down regulate at different times. *IiDIR*8 and *IiDIR*9 were nearly not expressed in roots, stems, leaves or flowers, but both of them were up regulated after treatment with MeJA. The regulation was lasting till the end of the experiment. This indicated that *IiDIR*8 and *IiDIR*9 may take part in defense response. *IiDIR*6/7/12/16/19 were lowly expressed in roots. After treatment with MeJA, they were up regulated at different times and last for a period of time.

## Discussion

DIR and DIR-like proteins belong to a multigene family. They are found in all of the major terrestrial plants [15] and are considered to have developed an important enzymatic reaction for the production of lignin and lignan during the time of the adaptation of aquatic plants to the terrestrial environment [31]. The gene number of DIR proteins in plants is different from each other. There are 25 DIRs in *A. thaliana*, 29 DIRs in B*. rapa* and 54 DIRs in rice [17, 19]. In this study, 19 DIRs are discovered from *I indigotica* by bioinformatics methods for the first time.

From the prediction of the ORFs, combined with the topology structures, it is found that the DIR amino acid sequences are divergent, ranging from the shortest protein of 183 aa (*Ii*DIR1) to the longest of 414 aa (*Ii*DIR19). The length of the DIR-e subfamily members is longer than DIRs from the other subfamilies; they range from 224 aa (*Ii*DIR18) to 414 aa (*Ii*DIR19). *Ii*DIRs in the DIR-a subfamily range from 183 aa (*Ii*DIR1) to 188 aa (*Ii*DIR2, *Ii*DIR3 and *Ii*DIR4). The length of the *Ii*DIRs in the DIR-b/d subfamily is similar with DIR-a subfamily, ranging from186 aa (*Ii*DIR5 and *Ii*DIR6) to 191 aa (*Ii*DIR12).

Sequence analysis of *Ii*DIRs, using currently available web-based bioinformatics tools (http://www.cbs.dtu.dk), indicated that most *Ii*DIRs have cleavable N-terminal signal peptides varying from 20 aa to 30 aa (Additional file 4) suggesting an extracellular localization.This means that these *Ii*DIRs are likely to be secreted proteins. N-glycosylation sites (Asn) are a feature of secreted proteins and have been found in *Fi*DIR1, the first and best characterized DIR protein [32]. Thirteen of these 19 *Ii*DIRS have more than one Asn sites, also indicating that most *Ii*DIRs were likely to be secreted protein.

The secondary and tertiary structures show that the *Ii*DIRs are all β-strand proteins. All the *Ii*DIRs have potential β-strands, separated by regions of coils. The β-strands shape the *Ii*DIR proteins like a barrel. The only α-helix exists in the *N*-terminus and appears to be the signal peptide. Both NetSurfP and PSIPRED prediction for the secondary structure of *Ii*DIRs show that DIRs are all-beta strand proteins. This is in agreement with previous studies [11]. Halls et al. [14] find out that the (+)-pinoresinol-forming dirigent protein from *F. intermedia* has been confirmed by circular dichroism analysis and is primarily composed of β-sheet and loop structures.

Hitherto, DIRs have not been crystallized [11], and X-ray or NMR structures are remain unavailable. Homologous proteins with known structures can serve as templates for modeling of DIRs. Therefore Phyre2 is used with intensive modeling to perform the prediction of the tertiary structures of *Ii*DIRs, as well as searching for the homologous proteins of *Ii*DIRs. It results that all the *Ii*DIRs are barrel-like proteins.

The topological tree (Figure 3) showed that *Ii*DIRs are divided into DIR-a, DIR-b/d, and DIR-e subfamilies. *P*DIR17 is separated from DIR-b/d subfamily and clustered to DIR-g subfamily. *Os*DIR1/2/3/4/9/49 from DIR-g subfamily are clustered with DIR-b/d subfamily. This is in agreement with Ralph’s studies [16, 17]. Ralph found subsequently that several sequences from the previous distinct DIR-d cluster were merged with the former DIR-b subfamily to form the new DIR-b/d subfamily and left the rice DIR-like proteins from the former DIR-d subfamily group to be a separate subfamily, DIR-g [17]. In this study, members of the DIR-b/d and DIR-g subfamilies are recombined again. This might be the result of extended DIR genes.

It should be noted that the transcript abundance of most *IiDIRs* are comparatively higher in roots and flowers than in stems and leaves. This is in accordance with Arasan’s finding in *B. rapa* DIRs [19]. It is well known that DIR genes are participate in lignin biosynthesis. So the *IiDIRs* transcript abundance in an organ specific manner in this study suggests that *IiDIRs* take possible roles in specific organs through lignin formation and participate in *I. indigotica’*s developmental processes. These organs also share characteristics that make them particularly prone to other stresses and protect themselves against stress attack. To find out *IiDIRs* transcript abundance in roots at the stress of MeJA, differential expression of *IiDIR* genes is mined from *I. indigotica* expression profiling database [18]. It shows that *IiDIR*8 and *IiDIR*9 may take part in defense response, because they are nearly not expressed in roots, stems, leaves or flowers, but both of them are up regulated after treatment with MeJA and the regulation lasting till the end of the experiment.

## Conclusions

In this study, 19 DIRs were distinguished from the *I. indigotica* transcription profiling database for the first time. Sequence characters and transcript abundance of these 19 full-length *Ii*DIRs were analyzed, respectively. The results showed that *Ii*DIR1 and *Ii*DIR2 are similar with *At*DIR5 and *At*DIR6. They might have the ability to produce (−)-lignans. The organ specific expression results in higher expression in roots and flowers than in stems and leaves indicated that roots and flowers may synthesis more lignin during plant development. *Ii*DIR6/7/8/9/12/16/19 were up regulated after treatment with MeJA, suggesting that they may take part in defense response. All this would provide basic data necessary for further studies.

## Methods

### Discovery of *IiDIRs* from the transcription profiling database

After 454 pyrosequencing of *I. indigotica* transcription profiling, a paired-end Solexa sequencing was carried out to maximize the sequence diversity. All of the data was assembled and provided a new database for the discovery of *IiDIRs*. The database was consisted of 65,196 unigenes at an average length of 1,503 bp. The largest unigene was 20,383 bp long while the length of the smallest unigene was 351 bp. Among all the unigenes, 30,131 genes was annotated [18].

In order to obtain all of the sequences of the DIRs in *I. indigotica* database, 1715 protein sequences, 1047 nucleotide sequences and 193 EST records of DIRs of other plants from the National Center for Biotechnology Information (http://www.ncbi.nlm.nih.gov/) were downloaded using the search word “dirigent”. All of the 2955 sequences were used as queries to search the *I. indigotica* transcription profiling database through basic local alignment search tool (TBLASTN or BLASTN) to determine all of the candidate *IiDIR* sequences with e value 1e-5. After removing sequences with alignment length less than 500 bp (the gene length of DIR was longer than 550 bp), there were 314 candidate *IiDIR* sequences. Since using the search word “dirigent” had not return dirigent proteins exclusively, all of the 314 *IiDIR* sequences were BLAST with NCBI using default parameters to remove the none dirigent sequences. The NCBI BLAST result was used to search the *I. indigotica* database again to mine the omission sequences of *Ii*DIRs.

To verify the reliability of the candidate *IiDIRs*, simple modular architecture research tool (SMART, http://smart.embl-heidelberg.de/) [20] was used to find the dirigent domain in these *IiDIRs* amino acid sequences respectively using default parameters.

### Sequence analysis

All the DIR cDNA sequences mining from the database were analyzed for their basic characteristics. NCBI Open Reading Frame Finder (ORF Finder) (http://www.ncbi.nlm.nih.gov/gorf/gorf.html) and Vector NTI Advance (TM) 11.0 were used to identify the whole ORF of each sequence. Predictions for MW and *p*I were performed using the entire ORFs on Vector NTI Advance (TM) 11.0. The TargetP 1.1 program accessible at http://www.cbs.dtu.dk/services/TargetP/[21] and the WoLF PSORT server (http://www.genscript.com/psort/wolf_psort.html) [22] were used to predict presence of N-terminal signal peptides and localization of the mature protein. The molecular formula of each *Ii*DIR protein was predicted by ProtParam (http://web.expasy.org/protparam/). Multiple protein sequences alignments of the *Ii*DIRs and *Br*DIRs were made with ClustalX 2.1. All the analysis was carried out using default settings.

### Prediction of the secondary and tertiary structures of *Ii*DIRs

Secondary structure predictions of the sequences were performed by NetSurfP (http://www.cbs.dtu.dk/services/NetSurfP/) [25], PSIPRED (http://bioinf.cs.ucl.ac.uk/psipred/) and Phyre2 (http://www.sbg.bio.ic.ac.uk/phyre2/html/page.cgi?id=index) [26] using default parameters. The prediction of the tertiary structures was carried out on Phyre2 with an intensive modeling. The same program was also used to search for homologs of *Ii*DIRs. The amino acid sequences of *Ii*DIRs were used as the target sequences.

### Computation of pairwise distances and phylogenetic analysis

Sequence similarities among the 19 full-length amino acids were computed by MEGA 5.05 with p-distance. All of the phylogenetic trees were built using MEGA 5.05 with 1000 bootstrap replicates. CONSENSE, also from MEGA 5.05, was used to create a consensus tree. Bootstrap values above 50% were added to the trees generated from the original data set. The ML tree of 197 DIRs was built using iTOL (http://itol.embl.de/) [33].

### Plant materials

The plant of *I. indigotica* was grown in the botanical garden of Second Military Medical University, Shanghai, China, and identified by Professor Hanming Zhang. Fresh roots, stems, leaves and flowers of this plant were harvested, frozen immediately in liquid nitrogen, and stored at −80°C for RNA isolation.

### Preparation of RNA and cDNA

Total RNA of *I. indigotica* was extracted from stored roots, stems, leaves and flowers respectively using the TIANGEN TRNzol-A^+^ Reagent for total RNA Isolation Kit (TIANGEN BIOTECH (BEIJING) CO., LTD, Beijing, China). The integrity of the RNA was visualized on ethidium bromide stained agarose gels, and the purity of the RNA was determined by UV spectrometry. The first-strand cDNA was reverse transcribed following the TransScript First-Strand cDNA Synthesis SuperMix’ User Manual (TransGen Biotech, Beijing, China).

### Real-time PCR

Real-time PCR was conducted on an ABI 7500 PCR system (Applied Biosystems, USA) using Fast SYBR^®^ Green Master Mix (Applied Biosystems) according to the manufacturer’s instructions. Reaction mixtures contained 1.5 μL of cDNA as template, 0.5 pmol of each primer and 10 μL of 2× Fast SYBR^®^ Green Master Mix in a final volume of 20 μL. Gene-specific primers (Additional file 6) for each *IiDIR* were designed through Primer Express 3.0 (Applied Biosystems). Specificity of each primer pair was checked by BLASTN searches against the *I. indigotica* RNA sequences to confirm designed primers were dirigent specific. Primer specificity (single product of expected length) was confirmed by analysis on a 0.8% agarose gel and by melting curve analysis. Gene *actin* was served as a quantification control. It was the best hit gene found in *I. indigotica* transcription profiling database through BLAST using 22 *A. thaliana*’s *actin* genes [NM_114519.2, NM_179953.2, NM_125328.3, NM_121018.3, NM_115235.3, NM_112764.3, NM_001085300.1, NM_001036427.2, NM_180280.1, NM_103814.3, NM_129772.1, NM_180032.1, AY114679.1, AY062702.1, AY120779.1, AK230311.1, U39480.1, U39449.1, U42007.1, U41998.1, AF308778.1 and NM_112046.3]. Primers for *I. indigotica actin* were also listed in Additional file 6.

The program for all real-time PCR reactions was: hold at 95°C for 20 s; 40 cycles of 3 s at 95°C and 30 s at 60°C. Data were analyzed using ABI 7500 sds Real-Time PCR system software (Applied Biosystems). All PCR reactions consisted of 3 technical replicates. Transcript abundance of each *IiDIR* gene was normalized to *actin* as control and compared with root as reference using 2^-△△Ct^ method.

### Transcript abundance of *IiDIRs* in *I. indigotica* hairy roots treated with MeJA

To get insight into the *IiDIRs’* transcript abundance induced with MeJA, the Illumina RNA-Seq data provide by Chen [18] was utilized. The RNA-Seq expression profile data were generated using the Illumina HiSeq™ 2000 platform, and included the hairy roots of *I. indigotica* treated with MeJA at 0, 1, 3, 6, 12 and 24 h. 0 h was used as control to normalize the expression level of other times. Finally, the heat map was constructed using the log2 transformed and normalized expression level data in MultiExperiment Viewer (MeV) [34].

## Availability of supporting data

Sequence data from this article can be found in the GenBank data libraries under accession numbers: *Ah*DIR1, AAZ20288.1; *As*DIR1, AAY41607.1; *At*DIR1, ABR46205.1; *At*DIR10, AAU90058.1; *At*DIR11, AAQ65106.1; *At*DIR12, AEE82982.1; *At*DIR13, AAP88352.1; *At*DIR14, AEE82984.1; *At*DIR15, AEE86966.1; *At*DIR16, AAP37695.1; *At*DIR17, CAB67637.1; *At*DIR18, AEE83298.1; *At*DIR19, AAO39937.1; *At*DIR2, AAP37801.1; *At*DIR20, AAU15178.1; *At*DIR21, AEE34435.1; *At*DIR22, AAU15153.1; *At*DIR23, AAT71988.1; *At*DIR24, AEE79355.1; *At*DIR25, AAP49521.1; *At*DIR3, AED95765.1; *At*DIR5, AAQ65109.1; *At*DIR6, AEE84795.1; *At*DIR7, AAQ89609.1; *At*DIR8, AEE75389.1; *At*DIR9, AAR20779.1; *At*DIRD4, AEC07124.1; *Fi*DIR1, AAF25357.1; *Fi*DIR2, AAF25358.1; *Gb*DIR1, AAS73001.2; *Gb*DIR2, AAY44415.1; *Hv*DIR1, AAA87042.1; *Hv*DIR2, AAA87041.1; *Hv*DIR3, AAB72098.1; *Nb*DIR1, BAF02555.1; *Os*DIR1, BAF20623.1; *Os*DIR10, BAF12227.1; *Os*DIR11, BAF22309.1; *Os*DIR12, BAF22310.1; *Os*DIR13, BAF22318.2; *Os*DIR14, BAC19943.1; *Os*DIR15, BAF22323.1; *Os*DIR16, BAC16397.1; *Os*DIR17, AAM74352.1; *Os*DIR18, BAD25846.1; *Os*DIR19, BAF13568.1; *Os*DIR2, BAF20624.1; *Os*DIR20, AAO17346.1; *Os*DIR21, BAB64642.1; *Os*DIR22, BAD52647.1; *Os*DIR23, BAF26452.2; *Os*DIR24, BAD53304.1; *Os*DIR25, AAM74358.1; *Os*DIR26, AAM74346.1; *Os*DIR27, BAD03849.1; *Os*DIR28, BAD03720.1; *Os*DIR29, BAD03711.1; *Os*DIR3, BAF20622.1; *Os*DIR30, BAD03854.1; *Os*DIR31, BAF29307.1; *Os*DIR32, BAF27737.1; *Os*DIR33, BAF27733.1; *Os*DIR34, AAX96293.1; *Os*DIR35, BAF27735.1; *Os*DIR36, BAF27734.1; *Os*DIR37, BAF29386.1; *Os*DIR38, BAF29514.1; *Os*DIR39, BAF29458.2; *Os*DIR4, BAF23585.1; *Os*DIR40, BAF29387.1; *Os*DIR41, BAF29454.1; *Os*DIR42, ABA94701.1; *Os*DIR43, BAH95407.1; *Os*DIR44, BAB89759.1; *Os*DIR46, BAH92863.1; *Os*DIR47, BAF27863.1; *Os*DIR48, BAF27866.1; *Os*DIR49, BAF27867.1; *Os*DIR5, BAF26451.1; *Os*DIR50, BAF23524.2; *Os*DIR51, BAD89460.1; *Os*DIR52, AAX96290.1; *Os*DIR53, ABA93522.1; *Os*DIR54, AAX96314.1; *Os*DIR6, BAF22196.1; *Os*DIR7, BAF22195.2; *Os*DIR8, BAB89617.1; *Os*DIR9, BAF20620.1; *P*DIR1, ABD52112.1; *P*DIR10, ABD52121.1; *P*DIR11, ABD52122.1; *P*DIR12, ABD52123.1; *P*DIR13, ABD52124.1; *P*DIR14, ABD52125.1; *P*DIR15, ABD52126.1; *P*DIR16, ABD52127.1; *P*DIR17, ABD52128.1; *P*DIR18, ABD52129.1; *P*DIR19, ABD52130.1; *P*DIR2, ABD52113.1; *P*DIR20, ABR27716.1; *P*DIR21, ABR27717.1; *P*DIR22, ABR27718.1; *P*DIR23, ABR27719.1; *P*DIR24, ABR27720.1; *P*DIR25, ABR27721.1; *P*DIR26, ABR27722.1; *P*DIR27, ABR27723.1; *P*DIR28, ABR27724.1; *P*DIR29, ABR27725.1; *P*DIR3, ABD52114.1; *P*DIR30, ABR27726.1; *P*DIR31, ABR27727.1; *P*DIR32, ABR27728.1; *P*DIR33, ABR27729.1; *P*DIR34, ABR27730.1; *P*DIR35, ABR27731.1; *P*DIR4, ABD52115.1; *P*DIR5, ABD52116.1; *P*DIR6, ABD52117.1; *P*DIR7, ABD52118.1; *P*DIR8, ABD52119.1; *P*DIR9, ABD52120.1; *Pp*DIR1, AAK38666.1; *Ps*DIR1, AAD25355.1; *Ps*DIR2, AAB18669.1; *Sb*DIR1, AAM94289.1; *Sb*DIR2, ABI24164.1; *Si*DIR1, AAT11124.1; *So*DIR1, AAR00251.1; *So*DIR2, CAF25234.1; *So*DIR3, AAV50047.1; *Ta*DIR1, AAC49284.1; *Ta*DIR2, AAM46813.1; *Ta*DIR3, BAA32786.3; *Ta*DIR4, AAR20919.1; *Tan*DIR1, ABE73781.1; *Th*DIR1, AAF25367.1; *Th*DIR2, AAF25368.1; *Tp*DIR, AAF25364.1; *Tp*DIR1, AAF25359.1; *Tp*DIR2, AAF25360.1; *Tp*DIR3, AAF25361.1; *Tp*DIR4, AAF25362.1; *Tp*DIR5, AAF25363.1; *Tp*DIR7, AAF25365.1; *Tp*DIR8, AAF25366.1; *Tp*DIR9, AAL92120.1; and *Zm*DIR1, AAF71261.2.

## Electronic supplementary material

Additional file 1: **Sequences of 19** ***IiDIR*** **s.** (TXT 14 KB)

Additional file 2: **Homology analysis of** ***IiDIRs.*** ^1^Disease resistance-responsive family protein. ^2^Disease resistance-responsive (dirigent-like protein) family protein. ^3^Defense response. ^4^Lignan biosynthetic process. (DOC 182 KB)

Additional file 3: **Gene characteristics of** ***DIRs*** **from** ***I. indigotica.*** (DOC 256 KB)

Additional file 4: ***Ii*** **DIRs secondary structure predictions.** *Ii*DIRs secondary structures were predicted with NetSurfP (http://www.cbs.dtu.dk/services/NetSurfP/) using the whole amino acid sequences. The probability calculated for three types of secondary structure is shown (dashed, α-helix; solid, β-strand; and dotted, coil) against the residue number of the *Ii*DIRs sequences. (DOCX 101 KB)

Additional file 5: **Maximum Likelihood (ML) phylogenetic trees of 19** ***Ii*** **DIRs.** The values on the branches are bootstrap proportions, which indicate the percentage values for obtaining this particular branching in 1000 repetitions of the analysis. The lengths of branches are proportional to evolutionary distances between species. -ln = 3970.33, model: WAG + F. (TIFF 640 KB)

Additional file 6: **Primer sequences used for real-time PCR.** (DOCX 30 KB)

Additional file 7: ***IiDIR*** **s’ Ct value of quantitative real-time PCR.** Ct values are presented as mean ± SEM, n = 3. (DOCX 30 KB)
