# Supplementary material for: The dirigent multigene family in Isatis indigotica: gene discovery and differential transcript abundance
Source: BMC Genomics. 2014 May 20;15(1):388. doi: 10.1186/1471-2164-15-388 (PMC4052678; doi:10.1186/1471-2164-15-388)
Supplement: Supplementary file 2 — Additional file 2: Homology analysis of IiDIRs. 1Disease resistance-responsive family protein. 2Disease resistance-responsive (dirigent-like protein) family protein. 3Defense response. 4Lignan biosynthetic process. (DOC 182 KB) [file 12864_2013_6080_MOESM2_ESM.doc]

**Additional file 2 Homology analysis of *IiDIRs***

| **Transcription profiling ID** | **Designate**  **name** | **Best matched clones** | **Query cover (%)** | **Identity (%)** | **Best homologous sp.** | **Functions** | **References** |
| --- | --- | --- | --- | --- | --- | --- | --- |
| comp13356_c0_seq1 | *IiDIR1* | XM_002887871.1 | 100 | 90 | *A. lyrata* subsp. *lyrata* | DRRP^1^ | Unpublished |
| comp33656_c0_seq1 | *IiDIR2* | EU186318.1 | 100 | 93 | *B. rapa* | DRRP | Unpublished |
| comp22738_c0_seq1 | *IiDIR3* | NM_117190.2 | 86 | 78 | *A. thaliana* | DLP^2^, DR^3^, LBP^4^ |  |
| comp22738_c0_seq2 | *IiDIR4* | NM_117190.2 | 86 | 79 | *A. thaliana* | DLP, LBP, DR |  |
| comp20562_c1_seq1 | *IiDIR5* | NM_123615.2 | 98 | 82 | *A. thaliana* | DLP, DR | Unpublished |
| comp20562_c1_seq2 | *IiDIR6* | NM_123615.2 | 99 | 83 | *A. thaliana* | DLP, DR | Unpublished |
| comp28741_c0_seq1 | *IiDIR7* | NM_105259.1 | 97 | 83 | *A. thaliana* | DLP, LBP, DR |  |
| comp28741_c0_seq2 | *IiDIR8* | NM_105259.1 | 97 | 83 | *A. thaliana* | DLP, LBP, DR |  |
| comp29140_c0_seq1 | *IiDIR9* | NM_112211.2 | 100 | 87 | *A. thaliana* | DLP, LBP, DR |  |
| comp33689_c0_seq1 | *IiDIR10* | EU186317.1 | 100 | 91 | *B. rapa* | DRRP | Unpublished |
| comp29669_c0_seq1 | *IiDIR11* | NM_179678.2 | 99 | 88 | *A. thaliana* | DLP, LBP, DR |  |
| comp32977_c0_seq1 | *IiDIR12* | NM_148075.1 | 65 | 84 | *A. thaliana* | DLP | Unpublished |
| comp26249_c0_seq1 | *IiDIR13* | NM_117432.3 | 98 | 90 | *A. thaliana* | DLP |  |
| comp26249_c0_seq2 | *IiDIR14* | NM_113307.2 | 100 | 92 | *A. thaliana* | DLP |  |
| comp26249_c0_seq3 | *IiDIR15* | XM_002870327.1 | 91 | 89 | *A. lyrata* subsp. *lyrata* | DRRP | Unpublished |
| comp14231_c0_seq1 | *IiDIR16* | XM_002876243.1 | 100 | 85 | *A. lyrata* subsp*. lyrata* | DLP | Unpublished |
| comp32662_c0_seq1 | *IiDIR17* | NM_129501.3 | 100 | 87 | *A. thaliana* | DLP, LBP |  |
| comp18321_c3_seq1 | *IiDIR18* | NM_202053.1 | 100 | 90 | *A. thaliana* | DLP |  |
| comp30687_c1_seq1 | *IiDIR19* | XM_002880957.1 | 63 | 88 | *A. lyrata* subsp. *lyrata* | DLP | Unpublished |

^1^ Disease resistance-responsive family protein

^2^ Disease resistance-responsive (dirigent-like protein) family protein

^3^ Defense response

^4^ Lignan biosynthetic process

**References**

1. Mayer K, Schüller C, Wambutt R, Murphy G, Volckaert G, Pohl T, Düsterhöft A, Stiekema W, Entian K-D, Terryn N: **Sequence and analysis of chromosome 4 of the plant Arabidopsis thaliana**. *Nature* 1999, **402**(6763):769-777.

2. Theologis A, Ecker JR, Palm CJ, Federspiel NA, Kaul S, White O, Alonso J, Altafi H, Araujo R, Bowman CL: **Sequence and analysis of chromosome 1 of the plant Arabidopsis thaliana**. *Nature* 2000, **408**(6814):816-820.

3. Kaul S: **Sequence and analysis of chromosome 2 of the plant Arabidopsis thaliana**. *Nature: International weekly journal of science* 1999, **402**(6763):761-768.

4. Erfle H, Ventzki R, Voss H, Rechmann S, Benes V, Stegemann J, Ansorge W, Zheng L, Cornel A, Wang R: **Sequence and analysis of chromosome 3 of the plant Arabidopsis thaliana**. *Nature* 2000, **408**(6814):820-822.
