# Supplementary material for: The dirigent multigene family in Isatis indigotica: gene discovery and differential transcript abundance
Source: BMC Genomics. 2014 May 20;15(1):388. doi: 10.1186/1471-2164-15-388 (PMC4052678; doi:10.1186/1471-2164-15-388)
Supplement: Supplementary file 3 — Additional file 3: Gene characteristics of DIRs from I. indigotica. (DOC 256 KB) [file 12864_2013_6080_MOESM3_ESM.doc]

***Additional file 3 Gene characteristics of DIRs from I.*** indigotica

| **Designate**  **name** | **Subfamily** | **ORF**  **(aa)** | **Transmembrane**  **region** | | **Domain** | | **Signal peptide**  **(aa)** | **N-Glyc(Asn) position** | **MW (kD)** | ***pI*** | **Location** | Molecular  formula |
| --- | --- | --- | --- | --- | --- | --- | --- | --- | --- | --- | --- | --- |
|  |  |  | **start** | **end** | **start** | **end** |  |  |  |  |  |  |
| *IiDIR1* | DIR-a | 183 | — | — | 34 | 178 | 23 | 54, 118 | 20.84 | 7.87 | Secreted | C_948_H_1436_N_230_O_270_S_11_ |
| *IiDIR2* | DIR-a | 188 | — | — | 39 | 183 | 29 | 59, 123 | 21.48 | 5.90 | Secreted | C_990_H_1470_N_234_O_273_S_11_ |
| *IiDIR3* | DIR-a | 188 | 13 | 30 | 39 | 183 | 29 | 59, 123 | 21.59 | 9.19 | Chloroplast | C_980_H_1492_N_248_O_274_S_11_ |
| *IiDIR4* | DIR-a | 188 | 13 | 30 | 39 | 183 | 29 | 3, 59, 123 | 21.40 | 9.30 | Chloroplast | C_973_H_1500_N_242_O_272_S_11_ |
| *IiDIR5* | DIR-b/d | 186 | — | — | 40 | 185 | 26 | 69, 128 | 20.36 | 9.10 | Secreted | C_922_H_1428_N_238_O_265_S_5_ |
| *IiDIR6* | DIR-b/d | 186 | — | — | 40 | 185 | 26 | 69, 128 | 20.35 | 6.57 | Secreted | C_926_H_1410_N_234_O_268_S_4_ |
| *IiDIR7* | DIR-b/d | 187 | — | — | 40 | 186 | 24 | 69, 170 | 20.18 | 9.74 | Secreted | C_913_H_1432_N_242_O_255_S_6_ |
| *IiDIR8* | DIR-b/d | 187 | — | — | 40 | 186 | 24 | 69, 170 | 20.17 | 9.85 | Secreted | C_914_H_1425_N_243_O_255_S_5_ |
| *IiDIR9* | DIR-b/d | 187 | — | — | 42 | 185 | 24 | 58, 70, 91, 126 | 20.48 | 9.64 | Secreted | C_918_H_1455_N_247_O_262_S_7_ |
| *IiDIR10* | DIR-b/d | 189 | — | — | 44 | 187 | 20 | 60, 72, 93, 128 | 20.80 | 9.16 | Secreted | C_939_H_1459_N_245_O_264_S_9_ |
| *IiDIR11* | DIR-b/d | 190 | — | — | 43 | 189 | 26 | 59, 144, 184 | 20.67 | 6.16 | Membrane | C_911_H_1443_N_239_O_277_S_12_ |
| *IiDIR12* | DIR-b/d | 191 | 5 | 27 | 36 | 179 | 30 | — | 21.07 | 5.56 | vacuolar | C_946_H_1464_N_250_O_279_S_5_ |
| *IiDIR13* | DIR-e | 268 | 27 | 49 | 127 | 267 | — | — | 28.09 | 5.93 | Chloroplast | C_1250_H_1963_N_329_O_379_S_10_ |
| *IiDIR14* | DIR-e | 244 | 7 | 29 | 103 | 243 | 24 | — | 25.37 | 4.87 | Secreted | C_1135_H_1771_N_295_O_341_S_8_ |
| *IiDIR15* | DIR-e | 268 | 27 | 49 | 127 | 267 | — | — | 28.05 | 5.93 | Chloroplast | C_1244_H_1958_N_330_O_382_S_9_ |
| *IiDIR16* | DIR-e | 315 | — | — | 152 | 311 | 21 | — | 33.42 | 4.79 | Vacuolar | C_1482_H_2317_N_397_O_470_S_3_ |
| *IiDIR17* | DIR-e | 317 | 5 | 22 | 158 | 314 | 20 | — | 33.14 | 5.01 | vacuolar | C_1462_H_2295_N_399_O_467_S_3_ |
| *IiDIR18* | DIR-e | 224 |  |  | 69 | 223 | — | 9 | 23.03 | 5.17 | Cytoplasmic | C_994_H_1557_N_279_O_326_S_9_ |
| *IiDIR19* | DIR-e | 414 | 7 | 24 | 259 | 413 | 21 | 194 | 39.94 | 5.05 | Chloroplast | C_1743_H_2720_N_484_O_568_S_9_ |
