# Supplementary material for: The dirigent multigene family in Isatis indigotica: gene discovery and differential transcript abundance
Source: BMC Genomics. 2014 May 20;15(1):388. doi: 10.1186/1471-2164-15-388 (PMC4052678; doi:10.1186/1471-2164-15-388)
Supplement: Supplementary file 4 — Additional file 4: Ii DIRs secondary structure predictions. IiDIRs secondary structures were predicted with NetSurfP (http://www.cbs.dtu.dk/services/NetSurfP/) using the whole amino acid sequences. The probability calculated for three types of secondary structure is shown (dashed, α-helix; solid, β-strand; and dotted, coil) against the residue number of the IiDIRs sequences. (DOCX 101 KB) [file 12864_2013_6080_MOESM4_ESM.docx]

**Additional file 4 *Ii*DIRs secondary structure predictions.** *Ii*DIRs secondary structures were predicted with NetSurfP (http://www.cbs.dtu.dk/services/NetSurfP/) using the whole amino acid sequences. The probability calculated for three types of secondary structure is shown (dashed, α-helix; solid, β-strand; and dotted, coil) against the residue number of the *Ii*DIRs sequences.
