# Supplementary material for: The dirigent multigene family in Isatis indigotica: gene discovery and differential transcript abundance
Source: BMC Genomics. 2014 May 20;15(1):388. doi: 10.1186/1471-2164-15-388 (PMC4052678; doi:10.1186/1471-2164-15-388)
Supplement: Supplementary file 6 — Additional file 6: Primer sequences used for real-time PCR. (DOCX 30 KB) [file 12864_2013_6080_MOESM6_ESM.docx]

| **Gene name** | **Gene ID** | **Forward primer (5'to3')** | **Reverse primer (5'to3')** |
| --- | --- | --- | --- |
| *Ii*DIR1 | comp13356_c0_seq1 | GACTTCTGCTGCCGTTACCAA | GCCACAGGTTCGGATTGATAG |
| *Ii*DIR2 | comp33656_c0_seq1 | GACTTCAATGCGTGGTTTTGC | TTGTCGGCTCCATCATCAAG |
| *Ii*DIR3 | comp22738_c0_seq1 | ATCGGTCCGAAGATCAATCG | TGTGGCATTAGCGGCGTTA |
| *Ii*DIR4 | comp22738_c0_seq2 | ACAACACGTGGATGGCTTTCA | TCTCTGGTCGGCTCCATCAT |
| *Ii*DIR5 | comp20562_c1_seq1 | GGAGAGAGCTGCCGATAATG | ACCACGGCATCTAAACCAAC |
| *Ii*DIR6 | comp20562_c1_seq2 | GAATCCGATAGTGTCCAAGGTGA | CCTGTGCATAGCCTCTAGCGAA |
| *Ii*DIR7 | comp28741_c0_seq1 | CGGAAGAGGATATGCACGAGC | CTCAACTACAGCATCTCCACTTG |
| *Ii*DIR8 | comp28741_c0_seq2 | CGGAAGAGGATATGCACAAGC | CTCAACCACAGCATTTCCACTC |
| *Ii*DIR9 | comp29140_c0_seq1 | CAAGGGATGTACGCAGGTGTT | TCGAGATCGTGCTTCCGTTA |
| *Ii*DIR10 | comp33689_c0_seq1 | GCAAACCCAACCTCCATCAT | TCGGAACTTCTGCCGTCAAG |
| *Ii*DIR11 | comp29669_c0_seq1 | GGCCATGAGCTTCTGTTTTGA | GCCGACGATAGGCATTTCTC |
| *Ii*DIR12 | comp32977_c0_seq1 | TCGTGGGATTGCCATGTTC | GACGTGAGCAGTGTGTGGAAA |
| *Ii*DIR13 | comp26249_c0_seq1_2 | AGCGCCATGTCTCATCTGTCT | AAGTGTTGTCCCGCAGGAAT |
| *Ii*DIR14 | comp26249_c0_seq2_3 | CCTTTCGCCAAGCAGATTG | GGTACACCGTTGATGCCGTTA |
| *Ii*DIR15 | comp26249_c0_seq3_2 | GCCATGTCGCATCTGTCAGT | TGCACCATCCACTTGATGCT |
| *Ii*DIR16 | comp14231_c0_seq1 | GACCCTTTCCTTGACCGTGTT | AGGCGTGAGAAGCCGTTCTAT |
| *Ii*DIR17 | comp32662_c0_seq1_3 | TGAACACGAGCCGTTACTTGA | TTCCGTTCACCTCGGTTTGT |
| *Ii*DIR18 | comp18321_c3_seq1-2 | TTTCACGGCCATGTTTGAGA | ACCCATGACCCCAAGATGAG |
| *Ii*DIR19 | comp30687_c1_seq1_1 | CGAATGGTGCAAACCTTCCT | TAGCCGTGGTTCCACCAAGT |
| *actin* | comp31788_c2_seq4 | ATCCTCCGTCTTGACCTTGCT | TTTCCCGTTCTGCTGTTGTG |

**Additional file 6 Primer sequences used for real-time PCR**
