# Supplementary material for: The dirigent multigene family in Isatis indigotica: gene discovery and differential transcript abundance
Source: BMC Genomics. 2014 May 20;15(1):388. doi: 10.1186/1471-2164-15-388 (PMC4052678; doi:10.1186/1471-2164-15-388)
Supplement: Supplementary file 7 — Additional file 7: IiDIR s’ Ct value of quantitative real-time PCR. Ct values are presented as mean ± SEM, n = 3. (DOCX 30 KB) [file 12864_2013_6080_MOESM7_ESM.docx]

**Additional file 7 *IiDIR*s’ Ct value of quantitative real-time PCR**

| **Gene** | **Root** | **Stem** | **Leaf** | **Flower** |
| --- | --- | --- | --- | --- |
| *Ii*DIR1 | 23.71±0.04 | 31.06±0.13 | 35.23±0.51 | 32.73±0.28 |
| *Ii*DIR2 | 22.29±0.04 | 27.91±0.02 | 26.76±0.05 | 19.67±0.156 |
| *Ii*DIR3 | 29.68±0.05 | 30.75±0.37 | 33.37±0.88 | 28.98±0.09 |
| *Ii*DIR4 | 28.99±0.16 | 28.38±0.51 | 31.84±1.05 | 26.29±0.04 |
| *Ii*DIR5 | 26.71±1.13 | 25.90±0.31 | 25.79±0.43 | 22.88±0.08 |
| *Ii*DIR6 | 34.39±0.13 | 33.35±0.48 | 28.52±0.07 | 30.67±0.01 |
| *Ii*DIR7 | 32.49±0.37 | 31.57±0.35 | 32.12±0.21 | 22.58±0.20 |
| *Ii*DIR8 | 35.23±0.69 | 35.04±1.79 | 34.54±0.61 | 32.11±0.33 |
| *Ii*DIR9 | 34.05±0.11 | 35.13±0.70 | 37.62±0.69 | 35.16±0.65 |
| *Ii*DIR10 | 22.52±0.10 | 24.70±0.04 | 22.92±0.04 | 19.42±0.12 |
| *Ii*DIR11 | 25.71±0.08 | 32.27±0.22 | 30.78±0.07 | 25.97±0.07 |
| *Ii*DIR12 | 32.79±0.31 | 31.87±0.50 | 34.34±0.43 | 32.53±0.19 |
| *Ii*DIR13 | 31.79±1.63 | — | — | 34.52±2.06 |
| *Ii*DIR14 | 29.64±0.04 | 31.62±0.43 | 30.96±0.08 | 28.12±0.07 |
| *Ii*DIR15 | 23.90±1.46 | 25.63±0.29 | 25.52±0.21 | 22.57±0.08 |
| *Ii*DIR16 | 33.66±0.23 | 33.82±0.16 | 35.28±0.62 | 34.00±0.16 |
| *Ii*DIR17 | 28.67±0.23 | 31.40±0.20 | 31.54±0.17 | 28.95±0.08 |
| *Ii*DIR18 | 27.92±0.14 | 29.23±0.19 | 28.12±0.16 | 25.74±0.08 |
| *Ii*DIR19 | 30.76±0.15 | 32.79±0.39 | 34.39±0.48 | 32.03±0.36 |
| *actin* | 17.68±1.75 | 18.58±1.55 | 16.63±1.79 | 16.57±1.75 |

Ct values are presented as mean±SEM, n=3
